# A robust and powerful GWAS method for family trios supporting within-family Mendelian randomization analysis

**DOI:** 10.21203/rs.3.rs-6163190/v1

**Published:** 2025-03-06

**Authors:** Shun Zhang, Hao-Wen Chen, Jia-Hao Mai, Qiu-Wen Zhu, Yuan-Sheng Li, Xian-Bo Wu, Ji-Yuan Zhou

**Affiliations:** Southern Medical University

**Keywords:** dynastic effect, residual population stratification, structure equation modelling, Mendelian randomization, family structure

## Abstract

Effect size estimates in genome-wide association studies (GWAS) and Mendelian randomization (MR) studies for independent individuals may be biased due to dynastic effect (DE) and residual population stratification (RPS). Existing GWAS methods for family trios effectively controlled such biases, while only using parental and offspring’s genotypes and offspring’s phenotype, and not incorporating parental phenotypes, which causes loss in estimation accuracy and test power. Therefore, we proposed a novel GWAS method based on structural equation modelling for family trios, denoted by FT-SEM. FT-SEM simultaneously uses parental and offspring’s genotypes and phenotypes. Simulation results demonstrate that FT-SEM substantially improves estimation accuracy and test power while controlling bias and type I error rate. Using family trios from Minnesota Center for Twin and Family Research (MCTFR), we found that DE and RPS greatly distort the results only based on independent individuals, and FT-SEM effectively corrects such biases. Combining the GWAS results from MCTFR with existing summary data, we performed several two-sample MR analyses. We observed that the effects of BMI on nicotine, alcohol consumption and behavior disorder were due to bias rather than causality. Our findings underscore the necessity of using families to validate the results of GWAS and MR, and highlight FT-SEM’s advantages.

## Introduction

**Figure 1:**
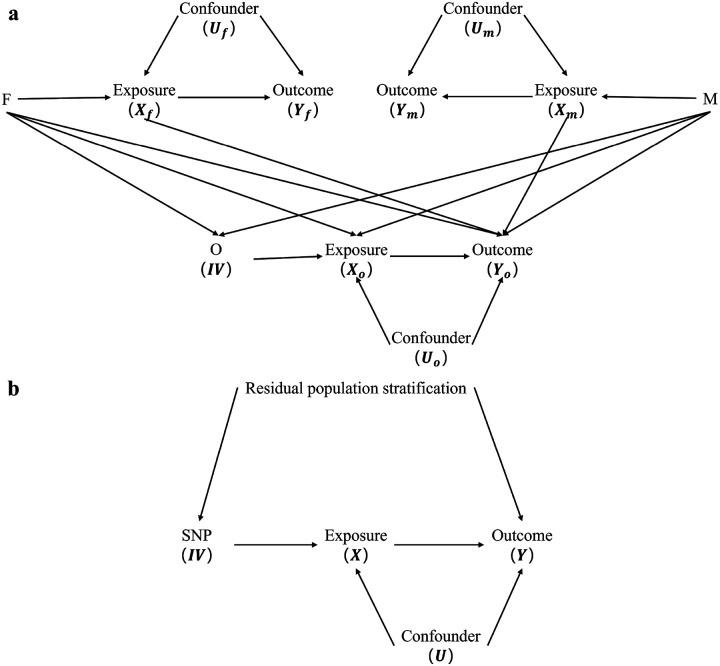
Directed acyclic graphs showing how dynastic effect and residual population stratification can confound GWAS and MR studies. The GWAS estimate of the genetic effect of the SNP on exposure or outcome and the MR estimate of the causal effect of the exposure on the outcome may be biased due to unobserved confounders among the SNP, the exposure, and the outcome. (a) Dynastic effect can induce a statistically confounding structure in the SNP-outcome association, where F, M and O are respectively the genotypes of the parents and their offspring in a family trio, $X_{f}$, $X_{m}$ and $X_{o}$, $Y_{f}$, $Y_{m}$ and $Y_{o}$, and $U_{f}$, $U_{m}$ and Uo are respectively the corresponding exposures, outcomes and confounders. (b) Residual population stratification can confound the association between the SNP and the outcome, which may bias the estimates of GWAS and MR, where X, Y and U are respectively the exposure, the outcome and the confounder.

Mendelian randomization (MR) is an approach that uses genetic variants (single nucleotide polymorphisms, SNPs) as instrumental variables (IVs) to estimate the causal effect between exposure and outcome^1-4^. MR has become increasingly popular due to the widespread application in the field of causal inference with summary data from genome-wide association studies (GWAS). For MR estimates to be valid, the genetic instrument should satisfy three key assumptions: (1) relevance, it must associate with the exposure, (2) independence, ensuring that no confounding factor influences both the instrument and the outcome, and (3) exclusion, the association between the instrument and the outcome must be entirely mediated via the exposure. However, an increasing number of studies have identified biases inherent in the traditional MR approaches when applied in practice. For instance, if the factors like dynastic effect (DE), residual population stratification (RPS), and horizontal pleiotropy, are not taken into account, biased estimates of causal effects may be obtained^5-7^. The issues of DE and RPS have been examined in Lawson et al.^8^ and Brumpton et al.^9,10^, which can cause bias in the MR analysis. Figure 1 illustrates these two mechanisms. In a family trio with both parents and their offspring, DE refers to the association between the parental genotypes and the offspring’s exposure/outcome which can be mediated via the unobserved parental phenotypes or other unknown mechanisms. The presence of DE (the arrows between parental genotypes and offspring’s exposure/outcome) violates the second key MR assumption of independence. Considering the parental genotypes will close these pathways and yield unbiased estimates. RPS represents that there are unobserved confounding factors which simultaneously affect genotype (IV) and outcome, thereby violating the second assumption of IVs. In addition, horizontal pleiotropy denotes a scenario where an IV not only influences the outcome indirectly through the exposure but also affects the outcome directly through other alternative pathways, which violates the exclusion assumption. Fortunately, horizontal pleiotropy could be corrected by the robust MR methods^7,11-16^.

On the other hand, confounding in GWAS estimates and causal estimates, which can be induced by DE and RPS, has been addressed by using family-based study designs^5,9,17-20^. Family-based study designs can generally be categorized into the following three types: (1) family trio, which includes genotypes and phenotypes of both parents and offspring, (2) mother-offspring pair, which includes genotypes and phenotypes of mother and her offspring, and (3) sibling pair, which includes genotypes and phenotypes of two sibs in a family. For family trios, a straightforward approach involves using the offspring’s phenotype as the dependent variable and the offspring’s genotype and parental genotypes as independent variables, and fitting a general linear model for GWAS^9^. This approach can be regarded as fitting a structural equation model (SEM) by only using observed variables. The resulting GWAS summary data can then be used for two-sample MR analysis. However, this approach does not use the phenotypic information of parents in family trios. For mother-offspring pairs, the mother’s genotype and phenotype, and offspring’s phenotype can be treated as observed variables, while the grandmother’s genotype and offspring’s genotype are modeled as latent variables within a SEM for GWAS^18-20^. If the grandmother’s or offspring’s genotype is available, it can also be included as observed variables in the model. This method corrects for biases arising from the maternal effect, such as maternal DE, and only requires maternal genotype, which enhances its applicability. However, this method ignores paternal effect, and discards paternal genotype and phenotype, and offspring’s genotype. As such, this method has mainly been used in specific research areas currently, such as studying birth weight. For sibling pairs, the genotypes and the phenotypes of two sibs can be differenced and then analyzed using a general linear model applied to the differenced data for GWAS. This method also corrects for DE and RPS and is widely used due to the relatively higher availability of sibling data in practice^21,22^. The two-sample MR methods could be conducted to get causal effect estimate by combining the GWAS summary data from this method. However, it is not applicable to family trios with only one offspring. Therefore, it is essential to further develop GWAS methods based on family trios to enhance data utilization while controlling for DE and RPS, and then facilitate the corresponding two–sample within-family MR analysis. In addition, in this study, for convenience, we used the offspring effect to refer to the effect of offspring’s genotype on his/her own phenotype. The paternal effect denotes the effect of paternal genotype on offspring’s phenotype, while the maternal effect represents the effect of maternal genotype on offspring’s phenotype.

Recall the SEM, as a diverse set of methods, which has been widely used to address problems in economics, sociology, psychology and epidemiology currently. Some researchers have applied SEM to analyze the family data for linkage analysis^23^, while others have modeled genes as latent variables, using SEM to perform multi-SNP and multi-phenotype association analyses^24^. In recent years, SEM has also been extended to accommodate the field of GWAS, such as the association analysis of five psychiatric traits, to take account of potential correlations among multiple phenotypes^25^. To address the inherent computational speed limitation of SEM when performing GWAS, an R package GW-SEM 2.0 has been developed^26^. Other researchers have suggested that incorporating the information on family structure into GWAS could give unbiased estimates of the effects between the SNP and phenotypes^5,9^. Using SEM to analyze mother-offspring pairs, some researchers divided the effects of SNP on birth weight into maternal and offspring effects^18^. With the widespread application of MR in recent years, concerns have been raised that MR analyses using independent individuals could derive biased causal effect estimates due to the factors such as DE and RPS^5,9^. To address these issues, two-sample MR methods based on family data^9^ and MR methods based on SEM using sibling data (MR-DoC) have been proposed^27^. The SEM applied to mother-offspring pairs is also considered to be combined with existing two-sample MR methods, providing more robust causal effect estimates^19^.

Therefore, in this study, we first constructed a SEM for family trios (denoted by FT-SEM) to conduct the family-based GWAS, which simultaneously uses parental genotypes and phenotypes as well as offspring’s genotypes and phenotypes in each family trio. We carried out extensive simulation studies to demonstrate the accuracy of point estimation and the precision of interval estimation of the genetic effect, and the corresponding hypothesis testing results obtained from GWAS using FT-SEM in the presence of DE and RPS. We also gave how to integrate the results of the GWAS using FT-SEM with existing two-sample MR methods for causal inference. We performed various simulation studies to illustrate the accuracy of point estimation and the precision of interval estimation of the causal effect, and the corresponding hypothesis testing results, even when DE and RPS exist. Utilizing 1,187 family trios from the Minnesota Center for Twin and Family Research (MCTFR) and summary data concerning 99,998 siblings derived from earlier studies^21^, we underscored the necessity of using families to validate the results of GWAS and MR, and highlighted FT-SEM’s advantages.

## Results

### Method overview

The proposed FT-SEM is a GWAS method for family trios, with its technical details described in the Methods section. FT-SEM considers parental genotypes and phenotypes as well as offspring’s genotypes and phenotypes as observed variables within the SEM framework. Additionally, it treats the genotypes of grandparents as latent variables, enabling a comprehensive analysis of family trios. After performing GWAS, the results obtained using FT-SEM can be organized as summary data and combined with the existing two-sample MR method, the ratio method^28^, for conducting two-sample MR analysis. Note that the ratio method can be considered as a special case of the inverse-variance weighted (IVW) method when there is only a single instrumental variable.

### Simulation overview

We performed simulation studies to evaluate the effectiveness of FT-SEM in GWAS and subsequent two-sample MR analysis, and compared it with existing GWAS methods based on family trios and those only based on independent individuals. In these comparisons, lm_parent represents the existing GWAS method for family trios, which fits a general linear model with the offspring’s phenotype being the dependent variable, the offspring’s genotype being the predictor and the parental genotypes being the covariates. This method is detailed in Brumpton et al.^9^ and it should be noted that lm_parent does not incorporate parental phenotypes. Additionally, lm denotes the GWAS method only based on the offspring in all the family trios (i.e. independent individuals), which fits a general linear model regarding the offspring’s phenotype as the dependent variable and the offspring’s genotype as a predictor. Note that lm ignores the information of all the parents in all the family trios.

In the GWAS simulation studies, we set the minor allele frequency (MAF) at a SNP to be 0.3 and considered the sample sizes of 1,000, 2,000, and 3,000 under three scenarios (DE, RPS, and there is no bias). Detailed simulation procedures and parameter settings are described in the Methods section. Furthermore, we used phenotypic variance explained (PVE) to specify the effect size. PVE indicates the proportion of variance in one variable explained by another, such as the proportion of variance in offspring’s phenotype explained by offspring’s genotype. The residual correlation between parental phenotypes and offspring’s phenotype was set to be 0.3 and 0.6 to indicate the within-family correlation.

In the simulation for two-sample MR analysis, we organized the GWAS results of offspring effect derived from the three methods (FT-SEM, lm_parent, and lm) as summary data and applied the ratio method to estimate the offspring causal effect for two-sample MR analysis. Before analysis, the sample was evenly divided into two subsets: one retaining SNPs and the exposure, and the other keeping SNPs and the outcome as observational data. We fixed the MAF at 0.3 and used the sample sizes of 2,000, 4,000, and 6,000, ensuring that the two samples in the MR analysis have equal sizes of 1,000, 2,000, and 3,000, respectively. Other simulation details are consistent with those in the GWAS simulation.

### Simulations for GWAS

**Figure 2:**
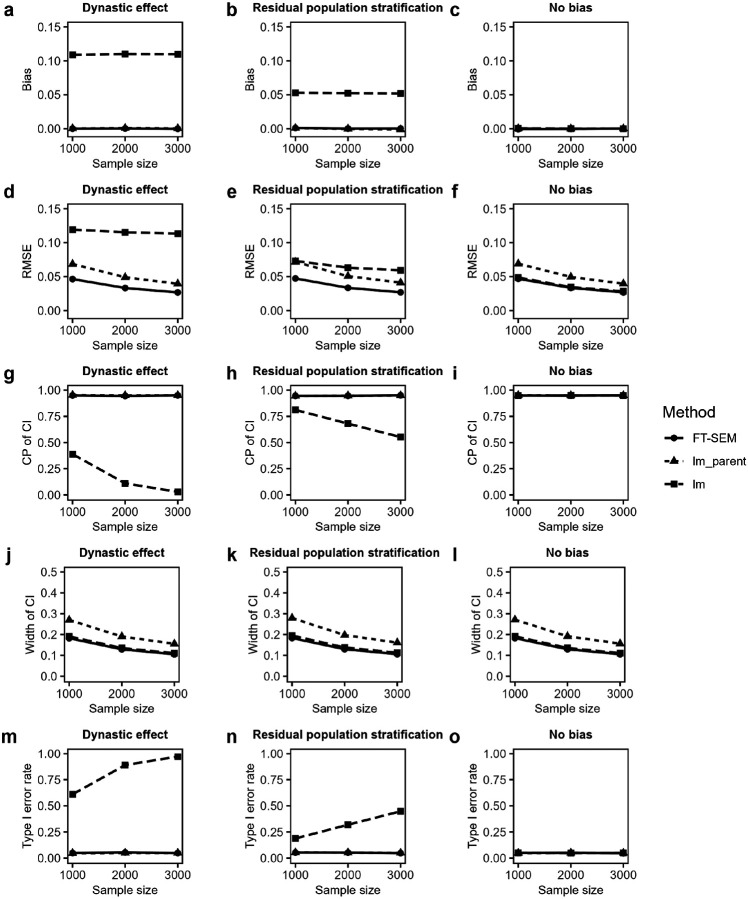
Simulated biases and RMSEs, CPs and average widths of the corresponding 95% CIs and type I error rates of offspring effect estimates for FT-SEM, lm_parent and lm with MAF of 0.3 when the sample size is fixed at 1,000, 2,000 and 3,000, with the PVE of offspring’s phenotype explained by offspring’s genotype being set to be 0, and the residual correlation being set to be 0.6 under dynastic effect, residual population stratification and no bias. **a-c** Biases of offspring effect estimates for FT-SEM, lm_parent and lm under dynastic effect, residual population stratification and no bias, respectively. **d-f**RMSEs of offspring effect estimates for FT-SEM, lm_parent and lm under dynastic effect, residual population stratification and no bias, respectively. **g-i**CPs of offspring effect estimates for FT-SEM, lm_parent and lm under dynastic effect, residual population stratification and no bias, respectively. **j-l**Average widths of offspring effect estimates for FT-SEM, lm_parent and lm under dynastic effect, residual population stratification and no bias, respectively. **m-o** Type I error rates of offspring effect estimates for FT-SEM, lm_parent and lm under dynastic effect, residual population stratification and no bias, respectively.

Figure 2 displays the simulated biases and root mean squared errors (RMSEs) of offspring effect estimates, the coverage probabilities (CPs) and the average widths of the corresponding 95% confidence intervals (CIs), and the type I error rates for FT-SEM, lm_parent and lm under the null hypothesis with the PVE of offspring’s phenotype explained by offspring’s genotype being set to be 0, and the residual correlation being set to be 0.6 under DE, RPS and no bias. First, we examined the performances of FT-SEM, lm_parent and lm in association analyses under DE. For point estimation, both FT-SEM and lm_parent provide unbiased estimates (the biases are close to 0), while lm yields biased estimates (the biases are far away from 0). The RMSEs of point estimates decrease as the sample size increases for all the three methods, while FT-SEM achieves the lowest RMSE, and the RMSE of lm_parent based on family trios is smaller than lm only based on independent individuals. For interval estimation, the CPs of FT-SEM and lm_parent are kept around 95% irrespective of sample size, whereas the CP of lm is not maintained close to 95% and decreases when the sample size increases. The widths of the CIs for all the three methods decrease when the sample size is larger, with FT-SEM and lm having similar performance and consistently producing the narrowest intervals. For hypothesis testing, both FT-SEM and lm_parent control the type I error rates well under the null hypothesis (around 0.05). However, lm has the inflated type I error rates. On the other hand, all the results under RPS are similar to those under DE. When no bias (e.g., no DE and no RPS) is present, all the methods obtain unbiased point estimates and precise confidence intervals, and control the type I error rates well. However, FT-SEM and lm have similar performances in RMSE and the average width of CI, which are less than those of lm_parent.

**Figure 3:**
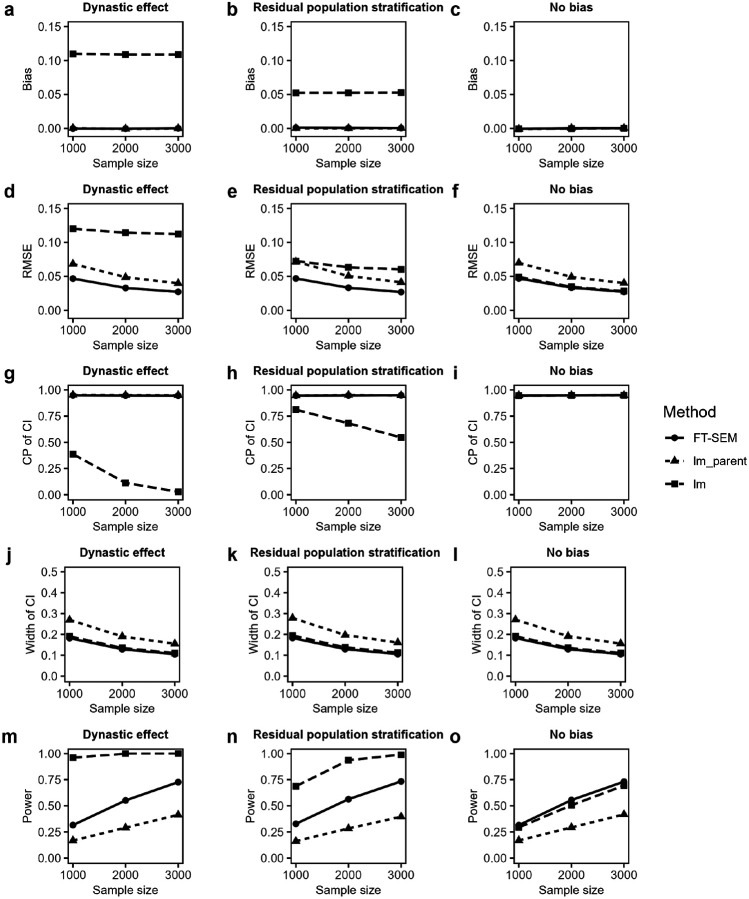
Simulated biases and RMSEs, CPs and average widths of the corresponding 95% CIs and powers of offspring effect estimates for FT-SEM, lm_parent and lm with MAF of 0.3 when the sample size is fixed at 1,000, 2,000 and 3,000, with the PVE of offspring’s phenotype explained by offspring’s genotype being set to be 0.2%, and the residual correlation being set to be 0.6 under dynastic effect, residual population stratification and no bias. **a-c** Biases of offspring effect estimates for FT-SEM, lm_parent and lm under dynastic effect, residual population stratification and no bias, respectively. **d-f** RMSEs of offspring effect estimates for FT-SEM, lm_parent and lm under dynastic effect, residual population stratification and no bias, respectively. **g-i** CPs of offspring effect estimates for FT-SEM, lm_parent and lm under dynastic effect, residual population stratification and no bias, respectively. **j-l** Average widths of offspring effect estimates for FT-SEM, lm_parent and lm under dynastic effect, residual population stratification and no bias, respectively. **m-o** Test power of offspring effect estimates for FT-SEM, lm_parent and lm under dynastic effect, residual population stratification and no bias, respectively.

Figure 3 illustrates the simulated biases and RMSEs of offspring effect estimates, CPs and average widths of the corresponding 95% CIs, and test powers for FT-SEM, lm_parent and lm under the alternative hypothesis, where the PVE in the offspring’s phenotype explained by the offspring’s genotype is fixed at 0.2% (i.e. the offspring effect is 0.069). The simulation results for point and interval estimations are consistent with those under the null hypothesis. For hypothesis testing, all the methods are more powerful for larger sample sizes. Under DE and RPS, FT-SEM demonstrates much higher power than lm_parent; lm has the largest power while lm cannot maintain its type I error rate. When no bias is present, FT-SEM still exhibits a little larger power than lm, and both FT-SEM and lm are much more powerful than lm_parent. This advantage of FT-SEM in power arises from its utilization of additional parental information, which is not considered in lm. In addition, Supplementary Figs. 1 and 2 show the corresponding results when the residual correlation is 0.3, which are similar to those for 0.6, except that, in the absence of bias, FT-SEM in the RMSE, the width of the 95% CI and the power performs slightly worse than lm.

### Simulations for two-sample MR

**Figure 4:**
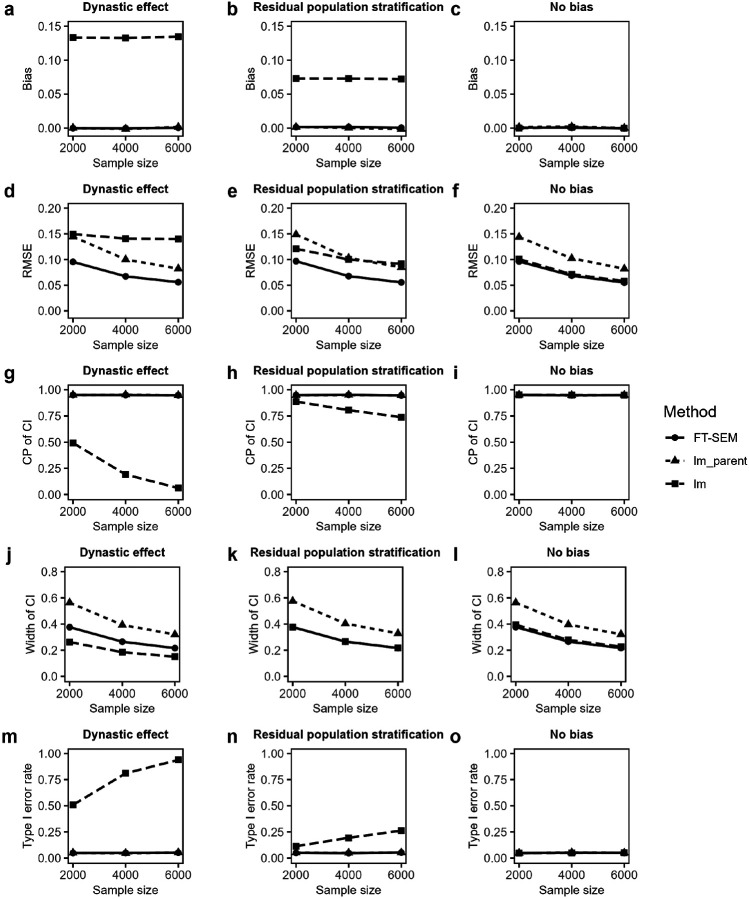
Simulated biases and RMSEs, CPs and average widths of the corresponding 95% CIs and type I error rates of causal effect estimates of offspring’s exposure on offspring’s outcome for FT-SEM, lm_parent and lm with MAF of 0.3 and the residual correlation of 0.6 under the null hypothesis when the sample size is fixed to be 2,000, 4,000 and 6,000. **a-c** Biases of causal effect estimates for FT-SEM, lm_parent and lm using the ratio method under dynastic effect, residual population stratification and no bias, respectively. **d-f** RMSEs of causal effect estimates for FT-SEM, lm_parent and lm using the ratio method under dynastic effect, residual population stratification and no bias, respectively. **g-i** CPs of causal effect estimates for FT-SEM, lm_parent and lm using the ratio method under dynastic effect, residual population stratification and no bias, respectively. **j-l** Average widths of causal effect estimates for FT-SEM, lm_parent and lm using the ratio method under dynastic effect, residual population stratification and no bias, respectively. **m-o** Type I error rates of causal effect estimates for FT-SEM, lm_parent and lm using the ratio method under dynastic effect, residual population stratification and no bias, respectively.

**Figure 5:**
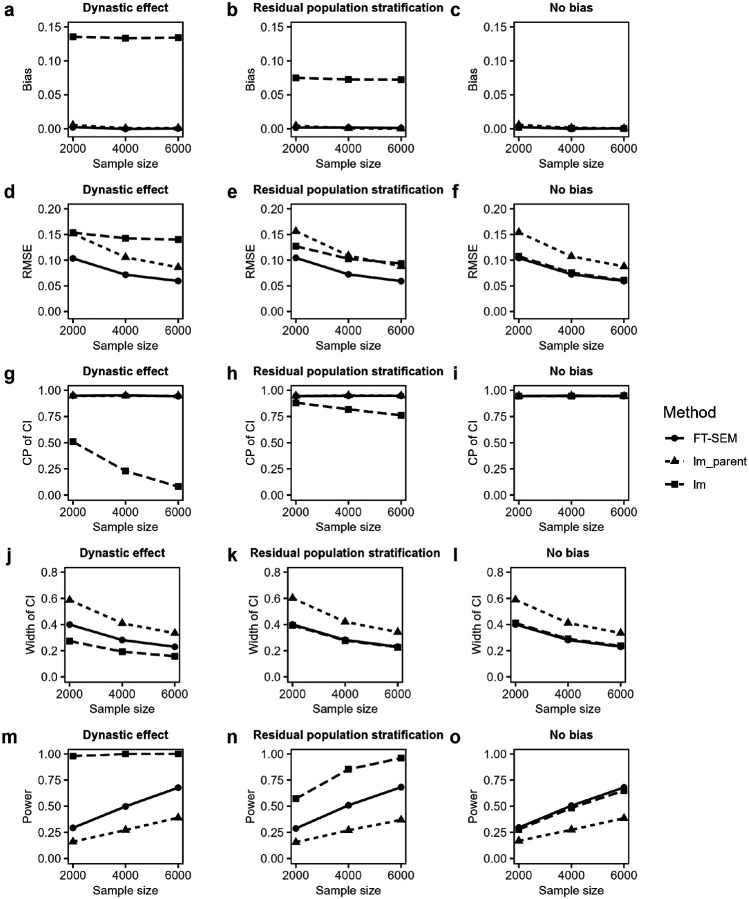
Simulated biases and RMSEs, CPs and average widths of the corresponding 95% CIs and powers of causal effect estimates of offspring’s exposure on offspring’s outcome for FT-SEM, lm_parent and lm with MAF of 0.3 and the residual correlation of 0.6 under the alternative hypothesis when the sample size is fixed to be 2,000, 4,000 and 6,000. **a-c** Biases of causal effect estimates for FT-SEM, lm_parent and lm using the ratio method under dynastic effect, residual population stratification and no bias, respectively. **d-f** RMSEs of causal effect estimates for FT-SEM, lm_parent and lm using the ratio method under dynastic effect, residual population stratification and no bias, respectively. **g-i** CPs of causal effect estimates for FT-SEM, lm_parent and lm using the ratio method under dynastic effect, residual population stratification and no bias, respectively. **j-l** Average widths of causal effect estimates for FT-SEM, lm_parent and lm using the ratio method under dynastic effect, residual population stratification and no bias, respectively. **m-o** Test power of causal effect estimates for FT-SEM, lm_parent and lm using the ratio method under dynastic effect, residual population stratification and no bias, respectively.

Figure 4 presents the simulated biases and RMSEs of causal effect estimates of offspring’s exposure on offspring’s outcome, CPs and average widths of the corresponding 95% CIs, and the type I error rates for FT-SEM, lm_parent and lm. Here, the PVE in offspring’s exposure explained by offspring’s genotype is taken to be 10% (i.e. the offspring effect is 0.488) to ensure that the SNP serves as a strong IV and the ratio estimate produces an accurate causal effect estimate. The PVE in offspring’s outcome explained by offspring’s genotype is set to be 0%, corresponding to a true causal effect value of 0 (i.e. the null hypothesis of no causal effect). From Fig. 4, we found that almost all the results are similar to those from previous GWAS. Specifically, FT-SEM achieves the lowest RMSE among all the methods while maintaining unbiased point estimates. It provides the narrowest interval widths while ensuring correct CPs and effectively controlling the type I error rates, except that the interval width of lm is the narrowest under DE. Figure 5 gives the corresponding results of estimations and test powers under the alternative hypothesis, where the PVE in offspring’s exposure due to offspring’s genotype is still fixed at 10% and the PVE in offspring’s outcome attributed to offspring’s genotype is assumed to be 0.2% (i.e. the true offspring causal effect is 0.141). It is shown in Fig. 5 that almost all the results are similar to those from previous GWAS under the alternative hypothesis. In detail, FT-SEM has the smallest RMSE among all the methods while obtaining unbiased point estimates. The interval widths of FT-SEM is the narrowest while keeping the CPs around 95%, except that lm has the narrowest interval width under DE. Furthermore, FT-SEM is much more powerful than lm_parent while FT-SEM can effectively control the type I error rate. In addition, Supplementary Figs. 3 and 4 demonstrate the corresponding results when the residual correlation is 0.3, with the conclusions similar to those for 0.6, except that, under no bias, FT-SEM in the RMSE, the width of the 95% CI and the power performs a little worse than lm.

### Real data applications

Firstly, we conducted a GWAS based on the MCTFR data set. This data set includes 6,784 individuals from 2,183 families, and 1,187 family trios with both parents and one offspring are selected for later analysis. We considered the following quality control (QC) criteria—(1) genotype call rate > 99%, (2) MAF > 1%, (3) individual call rate > 99%, and (4) Hardy-Weinberg equilibrium p-value > 1 × 10^−7^. As such, the data set retains 510,275 SNPs on autosomes. Moreover, the data set features five phenotypes: nicotine composite score (NIC), alcohol consumption composite score (CON), alcohol dependence composite score (DEP), illicit drug composite score (DRG), and behavioral disinhibition composite score (BD), which are highly correlated^29^. Hence, for the GWAS of these five phenotypes, a more lenient threshold of significance level, as proposed in the previous study^30^, is adopted as follows: a SNP locus is considered statistically significant if its p-value is less than 1 × 10^−5^ for one phenotype and its p-value is less than 1 × 10^−3^ for at least one additional phenotype. Furthermore, all the five phenotypes are continuous variables but deviate from normality assumption. To address this issue, we applied an inverse normal transformation (INT) as recommended in the previous study^31^ to convert them to approximate normality for later analysis.

In our real data applications, we compared the results of three methods (FT-SEM, lm_parent and lm). FT-SEM and lm_parent use 1,187 family trios, while lm utilizes only 2,183 independent individuals by randomly selecting one member from each family. Note that both FT-SEM and lm_parent can simultaneously estimate offspring, paternal and maternal effects, and having paternal or maternal effect means that there may be DE or RPS. In contrast, lm can only estimate offspring effect, which may be influenced by unadjusted paternal and maternal effects. Supplementary Tables 1–3 summarize the SNPs having the statistically significant offspring effects on NIC, CON, DEP, DRG and BD identified by FT-SEM, lm_parent and lm, respectively. It is noteworthy that FT-SEM and lm_parent jointly identify SNP rs9349391, which shows a significant association with DEP (p-value = 4.516 × 10^−6^) and a potential association with CON (p-value = 1.187 × 10^−4^) based on FT-SEM. It is also significantly associated with DEP (p-value = 8.980 × 10^−8^) and is potentially associated with CON (p-value = 2.923 × 10^−4^) and DRG (p-value = 9.212 × 10^−4^) based on lm_parent. However, no loci are identified simultaneously by all the three methods. This discrepancy may result from false positives in lm. Furthermore, Supplementary Tables 4 and 5 report the SNPs showing the statistically significant paternal effects associated with NIC, CON, DEP, DRG and BD revealed by FT-SEM and lm_parent, respectively. Among them, rs11012099, rs7965596 and rs4788008 are jointly detected by both methods. These loci are significantly associated with DEP, DEP and BD (p-values = 4.500 × 10^−6^, 3.050 × 10^−6^ and 6.810 × 10^−7^ respectively) and show potential associations with NIC, CON and DEP (p-values = 5.120 × 10^−6^, 2.000 × 10^−4^ and 4.810 × 10^−5^, respectively) based on FT-SEM. These loci also demonstrate significant associations with DRG, DEP and BD (p-values = 3.610 × 10^−7^, 7.920 × 10^−6^ and 9.660 × 10^−6^, respectively) and are potentially associated with DEP DRG and DEP (p-values = 8.600 × 10^−7^ 5.160 × 10^−4^ and 7.680 × 10^−4^, respectively) based on lm_parent. Moreover, Supplementary Tables 6 and 7 present significant loci for maternal effects observed by FT-SEM and lm_parent, respectively. Three loci (rs7299766, rs7309476 and rs2334242) are found by both methods. These loci show significant associations with CON, CON and BD (p-values = 5.950 × 10^−7^, 9.020 × 10^−7^ and 6.490 × 10^−7^, respectively) and potential associations with DEP, DEP and DRG (p-values = 4.990 × 10^−4^, 5.560 × 10^−4^ and 8.330 × 10^−7^, respectively) based on FT-SEM. These loci are also significantly associated with CON, CON and DRG (p-values = 5.750 × 10^−7^, 7.031 × 10^−7^ and 5.804 × 10^−7^, respectively), and demonstrate potential association with DEP, DEP and NIC (p-values = 1.437 × 10^−4^, 1.507 × 10^−4^ and 6.434 × 10^−7^ respectively) using lm_parent.

After the GWAS for the MCTFR data set, we performed two-sample MR analysis to investigate the causal effects of offspring’s body mass index (BMI) on offspring’s five phenotypes (NIC, CON, DEP, DRG and BD). For the first sample with BMI and genotypes, we used the GWAS summary data derived from the previous study based on sibling data^21^ with the sample size of 99,998 and independent individuals^32^ with the sample size of 681,275. The second sample consists of the GWAS summary data of offspring effects obtained from our previous analyses for the MCTFR data set. The IVW method serves as the primary approach for the two-sample MR analysis due to the presence of multiple instrumental variables. Specifically, IVW FT-SEM and IVW lm_parent refer to the methods where the sibling-based GWAS summary data are used as the first sample, and the GWAS summary data based on the MCTFR data set using FT-SEM and lm_parent, respectively, are used as the second sample. IVW lm is the approach where the GWAS summary data derived from independent individuals are employed as the first sample, and the GWAS summary data based on the MCTFR data set using lm are utilized as the second sample for MR analysis.

**Figure 6:**
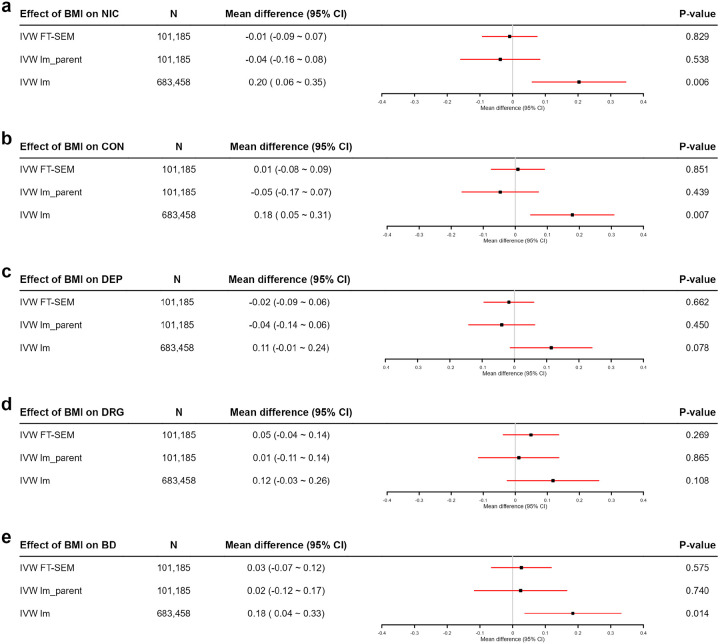
Point estimates, 95% CIs and *p*-values for the causal effects of offspring’s BMI on offspring’s NIC, CON, DEP, DRG and BD by using IVW FT-SEM, IVW lm_parent and IVW lm. The sample size for the two-sample MR is denoted as N, which represents the sum of the sample sizes used in the two stages. IVW FT-SEM utilizes sibling-based GWAS summary data for BMI and GWAS summary data derived from FT-SEM for NIC, CON, DEP, DRG and BD (N=101,185); IVW lm_parent is based on sibling-based GWAS summary data for BMI and GWAS summary data derived from lm_parent for the five outcomes (N=101,185); IVW lm uses GWAS summary data based on independent individuals for BMI and GWAS summary data derived from lm for the five outcomes (N=683,458).

Figure 6 presents the results of two-sample MR analysis. IVW lm identifies significant causal effects of offspring’s BMI on offspring’s NIC, CON and BD, with the p-values being 0.006, 0.007, and 0.014, respectively. On the other hand, the results of IVW lm show that causal effects of offspring’s BMI on offspring’s DEP and DRG are not statistically significant (p-values > 0.05). In contrast, both IVW FT-SEM and IVW lm_parent detect no causal effects of offspring’s BMI on offspring’s NIC, CON, DEP, DRG or BD (p-values > 0.05). The causal effects identified by IVW lm for NIC, CON and BD may result from DE or RPS, and thus may lead to false positives. In addition, IVW FT-SEM demonstrates the highest estimation precision (the narrowest interval widths), aligning with the findings from our simulation study.

Finally, as mentioned above, we only used the IVW method to conduct the two-sample MR analysis. Here, to further investigate whether our two-sample MR results could be attributed to horizontal pleiotropy, we also employed the weighted median, weighted mode and MR-Egger methods to carry out the two-sample MR analysis, which are robust against various forms of horizontal pleiotropy. Supplementary Table 8 summarizes the results of testing horizontal pleiotropy using the MR-Egger method, which shows that horizontal pleiotropy is not statistically significant in all the cases (p-values > 0.05). Supplementary Figs. 5–7 illustrate the results of the weighted median, weighted mode and MR-Egger methods, where the second sample is the GWAS summary data respectively derived from FT-SEM, lm_parent and lm for the five phenotypes in the MCTFR data set. These three figures show that offspring’s BMI does not exhibit significant causal effects on offspring’s five phenotypes, which is consistent with the conclusions drawn from the IVW method in Fig. 6 (except for the IVW lm results for NIC, CON and BD).

## Discussion

In this paper, we introduced a powerful method FT-SEM for GWAS which is based on SEM using family trios and further illustrated how to perform two-sample MR analysis utilizing the results derived from FT-SEM. FT-SEM leverages the information on both parental genotypes and phenotypes as well as offspring’s genotypes and phenotypes. Compared to previous methods based on family trios, FT-SEM uses a greater amount of information, thereby improving estimation accuracy and statistical power. Additionally, we explained how confounding factors arising from DE and RPS could bias MR studies for independent individuals. The simulation results indicate that when DE and RPS are present, the GWAS and MR methods based on independent individuals (i.e. lm and IVW lm) yield biased estimates and cannot control the type I error rates well. However, the GWAS methods based on family trios (i.e. FT-SEM and lm_parent) as well as the corresponding two-sample MR methods (IVW FT-SEM and IVW lm_parent) respectively based on the results of FT-SEM and lm_parent could provide unbiased point estimates and accurate CPs, and maintain well-controlled type I error rates in the presence of DE and RPS. Compared to lm_parent and IVW lm_parent, FT-SEM and IVW FT-SEM have lower RMSEs, narrower CIs, and greater statistical powers. In the GWAS for the MCTFR data set, we observed that the results of lm based on independent individuals are inconsistent with those of FT-SEM and lm_parent using family trios. This suggests that previous GWAS results only based on independent individuals might have been influenced by DE and RPS. We also conducted the family-based two-sample MR and the two-sample MR based on independent individuals using additional summary data^21,32^ and the GWAS summary data derived from FT-SEM, lm_parent and lm for the MCTFR data set to estimate the causal effects of BMI on NIC, CON, DEP, DRG and BD. We found that, after incorporating the paternal and maternal effects, there are no causal effects of offspring’s BMI on offspring’s NIC, CON and BD, which is consistent with the conclusions drawn from previous family-based MR analyses^9,21^, while IVW lm only based on independent individuals does. This demonstrates that the results of previous MR studies using independent individuals might have been subject to bias. On the other hand, the proposed FT-SEM method can be considered as an extension of SEM based on mother-offspring pairs^18^ to accommodate family trios. When the paternal effect is not considered, the model for FT-SEM is reduced to a SEM based on mother-offspring pairs. Furthermore, it should be noted in Brumpton et al.^9^ that another bias can affect GWAS analysis and two-sample MR analysis based on independent individuals—cross-trait assortative mating. Cross-trait assortative mating occurs when partners are selected based on different phenotypes. We also conducted the simulations to address this bias. The simulation results demonstrate that the model is robust to this bias (results not shown in the paper for brevity). In addition, our studies on the application of SEM in GWAS and MR further demonstrate the flexibility of SEM. In summary, our findings underscore the necessity of using families to validate the results of GWAS and MR and highlight the advantages of FT-SEM.

Sociological research indicated that there was a correlation between BMI and smoking^33^, but this relationship might be due to DE and RPS^21^. These effects operate through complex biological and social mechanisms, where genetic variants may exhibit pleiotropic effects on multiple phenotypes through shared neural pathways, and environmental factors also create shared familial contexts. Moreover, studies have showed that variations at genetic loci associated with alcohol addiction may influence the susceptibility to increased BMI during childhood and adolescence^34^. Our two-sample MR analyses in the real data applications suggest that the causal effects of offspring’s BMI on offspring’s phenotypes such as NIC, CON and BD may result from biases introduced by DE and RPS. For example, parental BMI may be correlated with offspring smoking and alcohol consumption, driven by family-related confounding factors such as a household culture that promotes these behaviors. Such a culture may lead to higher parental BMI and simultaneously increase the likelihood of smoking and alcohol consumption in offspring through multiple mechanisms: direct environmental exposure, behavioral modeling, social learning and the establishment of family norms. These mechanisms can be further modified by epigenetic regulation, where parental lifestyle factors may influence offspring’s phenotypes through DNA methylation and other epigenetic modifications. Since this confounder cannot be directly adjusted in the model, it manifests as an effect of parental BMI on offspring smoking and alcohol consumption. In our two-sample MR analyses based on independent individuals, parental SNPs influence offspring smoking and alcohol consumption via the parental BMI pathway and impact offspring’s SNPs through genetic inheritance. In this scenario, parental SNPs act as confounders, distorting the results of the two-sample MR analysis based on independent individuals—a phenomenon we term “dynastic effect”. The IVW FT-SEM and IVW lm_parent methods discussed in this study address this issue by including parental SNPs as covariates in the model. This inclusion blocks the confounding pathway, preventing bias from DE and producing unbiased causal effect estimates.

In general, the sample size available for family-based GWAS and two-sample MR analyses is smaller than GWAS and two-sample MR analyses based on independent individuals. Therefore, in practice, the results obtained from FT-SEM and IVW FT-SEM can be considered more robust but less efficient estimates compared to those from GWAS and two-sample MR methods using independent individuals. Consequently, if there is evidence of discrepancies between the estimates from the methods based on independent individuals and those based on family data, the robust and unbiased family-based estimates are generally preferred. While our proposed FT-SEM method is unaffected by DE and RPS when conducting GWAS analyses and using the corresponding GWAS results for two-sample MR analyses, it remains susceptible to horizontal pleiotropy. This issue could be addressed by incorporating robust two-sample MR methods that correct for or model horizontal pleiotropy^7,11-15^.

There are five potential limitations to our proposed FT-SEM method. Firstly, FT-SEM assumes multivariate normality among the observed variables and linearity in the relationship between genotypes and phenotypes. In instance of non-normality, employing an appropriate data transformation could aid in fulfilling the assumption of multivariate normality^31^. Secondly, we assumed additive genetic effects for all the genotypes and the genetic effects across the generations were equal. These assumptions are generally valid in biological contexts. However, if it is not met, the model’s estimates might be subject to some bias. Thirdly, in contrast to linear models, FT-SEM needs more computational time. Fourthly, FT-SEM is only applicable to the scenarios where each family has a single offspring. If a family has multiple offspring, the model needs to be extended or alternative methods should be considered. Lastly, the model assumes the presence of only main effects, without considering interactions.

In summary, we proposed a novel method in this paper, FT-SEM, based on the SEM using family trios to perform GWAS and further introduced how to use the results from FT-SEM to conduct two-sample MR analysis. Compared to the traditional methods for GWAS and two-sample MR using family trios, FT-SEM produces more precise estimates and more powerful tests, while ensuring robust estimation. Future work should focus on addressing how to enhance the computational speed of the SEM, using our proposed FT-SEM method to provide more robust summary data for subsequent downstream two-sample MR analyses and extending our methods to adapt to different family structures (e.g. nuclear families with multiple offspring and pedigrees). Additionally, efforts could be directed toward refining FT-SEM to address the scenarios involving nonlinear effects and interaction terms between variables in future.

## Methods

### FT-SEM method

In family trios, the genetic effects at single loci can be partitioned into paternal, maternal and offspring components (respectively denoted by $\beta_{FE}$, $\beta_{ME}$ and $\beta_{OE}$), which is similar to early researches^9,19^. Therefore, the SEM model (FT-SEM) for family trios can be represented as:

#(1) $$Z_{i}=\beta_{0}+G_{i}\beta +U_{i}+\epsilon_{i}$$

$$Z_{i}=\left(\;\begin{array}{c} Z_{F,i}\\ Z_{M,i}\\ Z_{O,i} \end{array}\;\right),\beta_{0}=\left(\;\begin{array}{c} \beta_{\;0F}\\ \beta_{\;0M}\\ \beta_{\;0O} \end{array}\;\right),G_{i}=\left[\begin{array}{ccc} GF_{1,i} & GM_{1,i} & F_{i}\\ GF_{2,i} & GM_{2,i} & M_{i}\\ F_{i} & M_{i} & O_{i} \end{array}\right],\beta =\left(\;\begin{array}{c} \beta_{\;FE}\\ \beta_{\;ME}\\ \beta_{\;OE} \end{array}\;\right),U_{i}=\left[\begin{array}{c} U_{F,i}^{T}\beta_{\mathrm{UE},F}\\ U_{M,i}^{T}\beta_{\mathrm{UE},M}\\ U_{O,i}^{T}\beta_{\mathrm{UE},O} \end{array}\right]\;\;\epsilon_{i}=\left(\;\begin{array}{c} \epsilon_{F,i}\\ \epsilon_{M,i}\\ \epsilon_{O,i} \end{array}\;\right)∼\mathrm{MVN}\left(0,\left[\begin{array}{ccc} \sigma_{F}^{2} & \sigma_{FM} & \sigma_{FO}\\ \sigma_{FM} & \sigma_{M}^{2} & \sigma_{MO}\\ \sigma_{FO} & \sigma_{MO} & \sigma_{O}^{2} \end{array}\right]\right)$$

where $Z_{F,i}$, $Z_{M,i}$ and $Z_{O,i}$ are respectively the phenotypes of the father, the mother and the offspring in family i(i=1,2,⋯,N), where N is the number of family trios (i.e. sample size). $\beta_{0F}$, $\beta_{0M}$ and $\beta_{0O}$ represent the intercept terms for the parents and their offspring, respectively. $F_{i}$, $M_{i}$, $O_{i}$, $GF_{1,i}$, $GM_{1,i}$, $GF_{2,i}$ and $GM_{2,i}$ are the numbers of the genetic variants respectively corresponding to the father, the mother, the offspring and the grandparents of the offspring in family i, even if $GF_{1,i}$, $GM_{1,i}$, $GF_{2,i}$ and $GM_{2,i}$ may not be collected in practice and will be regarded as latent variables in SEM later. $\beta_{FE}$, $\beta_{ME}$ and $\beta_{OE}$ are paternal, maternal and offspring effects. $U_{F,i}$, $U_{M,i}$ and $U_{O,i}$ are p-dimensional vectors representing the confounding variables for the parents and the offspring in family i, respectively. $\beta_{UE,F}$, $\beta_{UE,M}$ and $\beta_{UE,O}$ are p-dimensional vectors representing the corresponding effects of the confounding variables. $\epsilon_{i}$ represents the residual vector for family i, which follows a multivariate normal distribution, where $\epsilon_{F,i}$, $\epsilon_{M,i}$, and $\epsilon_{O,i}$ are respectively the residuals of the father’s, mother’s and offspring’s phenotypes. Here, $\sigma_{F}^{2}$, $\sigma_{M}^{2}$ and $\sigma_{O}^{2}$ are used to respectively denote the variances of the residuals of the father’s, mother’s and offspring’s phenotypes. Moreover, $\sigma_{FM}$, $\sigma_{FO}$ and $\sigma_{MO}$ are the covariances of the residuals among the father’s, mother’s, and offspring’s phenotypes, respectively. We assumed that the genetic effects in the parental generation are respectively identical to those in the offspring generation (i.e. sharing the same $\beta_{FE}$, $\beta_{ME}$ and $\beta_{OE}$). This assumption has been mentioned in previous study^18^.

**Figure 7:**
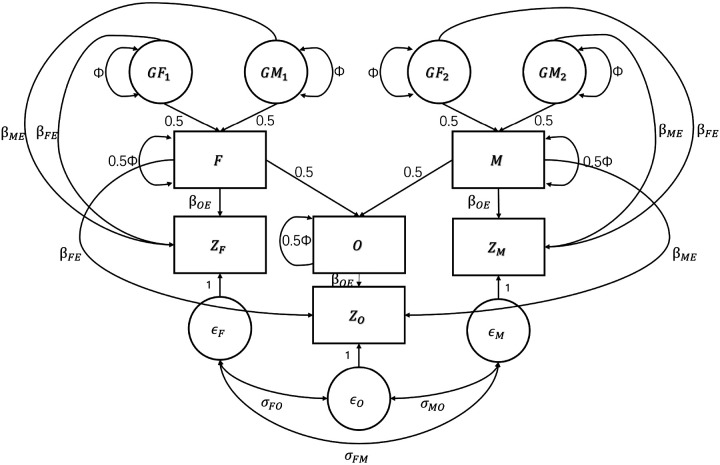
FT-SEM used to estimate paternal, maternal and offspring genetic effects on phenotypes. One-headed arrows represent causal paths and two-headed arrows indicate correlation relationships. The six observed variables (in squares) denote the father’s, mother’s and offspring’s genotypes (F, M and O) and their phenotypes ($Z_{F}$, $Z_{M}$ and $Z_{o}$). The latent variables (in circles) represent the genotypes of the grandparents (GF1, $GM_{1}$, $GF_{2}$ and $GM_{2}$). The total variances of the latent genotypes and those of the observed genotypes are set to be Φ and are estimated from the data. The path coefficients between the offspring and their parents are both set to be 0.5 according to Mendelian inheritance. The path coefficients $\beta_{FE}$, $\beta_{ME}$ and $\beta_{OE}$ refer to paternal, maternal and offspring effects on offspring’s phenotype, respectively.

This model can be fitted using the SEM. Figure 7 presents the path diagram of FT-SEM, which can be fitted using the maximum likelihood estimation with the OpenMx software package^35^. Hypothesis testing for FT-SEM is conducted using the Wald test. In this model, we simultaneously tested the paternal, maternal and offspring effects ($\beta_{FE}$, $\beta_{ME}$ and $\beta_{OE}$). The results can serve as summary data for various downstream analyses, including two-sample MR analysis. Particular importance is to test for the offspring effect ($\beta_{OE}$) and to conduct its corresponding two-sample MR analysis. In addition, some robust two-sample MR methods can be employed to control horizontal pleiotropy^7,11-15^. Furthermore, note that the estimated causal effect may be biased if the associations between SNPs and both the exposure and the outcome are estimated within the same sample^36^. Fortunately, this bias can be eliminated by employing a sample-splitting approach to estimate the associations in different samples. Finally, in the FT-SEM model, we assumed that there is no additional relationship between parental phenotypes and offspring phenotypes. This assumption is based on the fact that the effects of parental phenotypes on offspring’s phenotypes can be partly explained by parental genotypes, while the remaining effects could be reflected through considering the correlations between the residuals of parental phenotypes and that of offspring’s phenotype (i.e. $\sigma_{FO}$ and $\sigma_{MO}$). In fact, this assumption has also been made in previous structural equation models based on mother-offspring pairs^18^.

### Simulation settings

Firstly, we performed the simulations to investigate the bias and the RMSE of the point estimate of the offspring effect ($\beta_{OE}$), the CP and the average width of the corresponding 95% CI, and the type I error rate and the test power of FT-SEM in modeling family trios. We generated the genotypes of the grandparents (i.e. $GF_{1}$, $GM_{1}$, $GF_{2}$, and $GM_{2}$) based on MAF at a single SNP and assuming that Hardy-Weinberg equilibrium holds, and then simulated the genotypes of the parents and the offspring (i.e. F, M, and O) according to Mendelian inheritance. Each of the SNP effect sizes ($\beta_{FE}$, $\beta_{ME}$ and $\beta_{OE}$) and the confounding effects (i.e. $\beta_{UE,F}$, $\beta_{UE,M}$ and $\beta_{UE,O}$) was set by using the equation $\beta =\frac{PVE}{Var(SNP)}$, where PVE represents the proportion of the variance in the phenotype explained by the SNP, and Var(SNP) denotes the variance of the genotype distribution at the SNP. If $PVE_{sum}$ was used to represent the sum of the PVE values across all the variables, then the three variances of the residuals (i.e. $\sigma_{F}^{2}$, $\sigma_{M}^{2}$ and $\sigma_{O}^{2}$) were assumed to be the same for simplicity and were set by using the $PVE_{sum}$ (i.e. $\sigma_{F}^{2}=\sigma_{M}^{2}=\sigma_{O}^{2}=1-PVE_{sum}$). We used the parameter ρ to control the correlations among the residuals with $\sigma_{FM}=\sigma_{FO}=\sigma_{MO}=\rho \;(1-PVE_{sum})$, and ρ was set to be 0.3 and 0.6. The simulations for GWAS were divided into three scenarios: the presence of DE, the presence of RPS, and no bias. To simulate DE, we considered a homogenous population with MAF = 0.3 and assigned nonzero values to the parental effects ($\beta_{FE}$ and $\beta_{ME}$). Specifically, when $\beta_{FE}$ or $\beta_{ME}$ is nonzero, DE is present. In our simulations, we assumed that $PVE_{FE}=PVE_{ME}=0.5$, corresponding to $\beta_{FE}=\beta_{ME}=0.109$. To simulate RPS, we considered a population consisting of two subpopulations with MAFs being 0.25 and 0.35, respectively, while keeping other parameters to be the same in these two subpopulations which are both consistent with those under DE. As such, we introduced a variable L (assigned a value of 1 if the family trio belongs to the first subpopulation and 2 if the family trio belongs to the second subpopulation, where a family trio comes from these two subpopulations with equal probabilities) with its effect on the phenotype being denoted as $\beta_{LE}$. The PVE of this variable was set to be $PVE_{LE}=5$, corresponding to $\beta_{LE}=0.447$. In this scenario, we assumed no DE (i.e. $\beta_{FE}=\beta_{ME}=0$). To simulate no bias scenario, we considered a homogenous population and set $\beta_{FE}=\beta_{ME}=0$. For all the three scenarios simulated, the intercept was taken as 0 and we generated the values of the confounding variable U for the father, the mother and the offspring. These values of U for the parents and the offspring were independent of each other but shared the same $PVE_{U}=0.3$, corresponding to identical effects on the phenotypes (i.e. $\beta_{UE,F}=\beta_{UE,M}=\beta_{UE,O}=0.548$). Then, we evaluated all the methods under both the null and alternative hypotheses. Under the null hypothesis, we set $PVE_{OE}=0$, corresponding to $\beta_{OE}=0$. Under the alternative hypothesis, we set $PVE_{OE}=0.2$, corresponding to $\beta_{OE}=0.069$. After setting the parameters, the phenotypes were generated using Eq. (1). Once the phenotypes were generated, the genotypes of the grandparents and the variables L and U were removed and treated as missing values. We considered the sample sizes (N) of 1,000, 2,000 and 3,000, and compared the results of three methods (FT-SEM, lm_parent and lm). For each simulation setting, we considered 10,000 replicates.

As mentioned previously, after conducting the GWAS using FT-SEM based on family trios, the GWAS results can be applied to various downstream analyses, including two-sample MR. In this context, robust two-sample MR methods can be employed to consider horizontal pleiotropy. Therefore, we further performed the simulations to investigate the bias and the RMSE of the point estimate of the causal effect of offspring’s exposure on offspring’s outcome, the CP and the average width of the corresponding 95% CI, and the type I error rate and the test power of these two-sample MR methods. For simplicity, we selected the ratio estimation approach as the two-sample MR method, which can be considered as a simplified version of the IVW method with only one instrumental variable. In the two-sample MR simulation, the exposure and outcome variables (denoted as X and Y respectively) are simulated separately. Firstly, we set $\alpha =\frac{PVE_{gy}}{PVE_{gx}}$, where $PVE_{gx}$ represents the PVE of the exposure explained by offspring effect (similar to $PVE_{OE}$ in the GWAS) and $PVE_{gy}$ denotes the PVE of the outcome attributed to vertical pleiotropic effect in the absence of horizontal pleiotropy. Then, the generation of the exposure can follow Eq. (1), and the simulation of the outcome is also based on Eq. (1) with an additional term αX included to represent the causal effect of the exposure on the outcome. For simulating the exposure in all the scenarios, we set $PVE_{U}=20\%$ (i.e. $\beta_{UE,F}=\beta_{UE,M}=\beta_{UE,O}=0.447$) and $PVE_{gx}$ to be 10% for the exposure (i.e. $\beta_{OE}=0.488$), indicating that the SNP is a strong instrumental variable. For simulating the outcome in all the scenarios, we set $PVE_{U}=30\%$ (i.e. $\beta_{UE,F}=\beta_{UE,M}=\beta_{UE,O}=0.548$). Afte that, under the null hypothesis, we set $PVE_{gy}=0\%$, corresponding to α=0, and we assumed that $PVE_{gy}=0.2\%$, corresponding to α=0.141 under the alternative hypothesis. Furthermore, when simulating the outcome, the $PVE_{OE}$ was set to 0%, indicating that there are no horizontal pleiotropy. When simulating the exposure in the presence of DE, we considered a homogenous population with MAF=0.3 and specified $PVE_{FE}=PVE_{ME}=2.5\%$, which corresponds to $\beta_{FE}=\beta_{ME}=0.244$. For simulating the outcome in the presence of DE, we used the same population and set $PVE_{FE}=PVE_{ME}=0.4\%$, leading to $\beta_{FE}=\beta_{ME}=0.098$. For the simulations involving the exposure in the presence of RPS, we considered two subpopulations with MAFs being 0.25 and 0.35, respectively, which is similar to the simulation for GWAS. And then, we also introduced L in the model to perform the effect of RPS, and set $PVE_{LE}=5\%$, resulting in $\beta_{LE}=0.224$ to simulate the exposure. After that, when simulating the outcome in the presence of RPS, we assigned $PVE_{LE}=10\%$, which yields $\beta_{LE}=0.316$. In this scenario, we assumed no DE (i.e. $\beta_{FE}=\beta_{ME}=0$). In the scenario of no bias, for both the exposure and outcome variables, we considered a homogenous population which is the same as DE, and set $\beta_{FE}=\beta_{ME}=0$. After generating the exposure and outcome variables, the samples were randomly divided into two parts of equal sizes. In the first part (the first sample), the outcome variable was removed to investigate the association between the offspring’s genotype and the exposure, while in the second part (the second sample), the exposure variable was deleted to study the association between the offspring’s genotype and the outcome. For each simulation setting, we used the sample sizes of 2,000, 4,000, and 6,000, ensuring that the two samples in the MR analysis had equal sizes of 1,000, 2,000, and 3,000, respectively. Finally, we considered 10,000 replicates, and we compared the results of three methods (FT-SEM, lm_parent and lm) with the ratio estimation approach.

### Real data applications

To illustrate the approaches and evaluate the potential biases from DE and RPS, we performed the family-based GWAS by using the MCTFR data set, and combined these GWAS results and the summary data from the sibling-based GWAS^21^ to conduct the family-based two-sample MR analyses. The MCTFR data set is accessible via the Database of Genotypes and Phenotypes (dbGaP) with the accession number phs000620.v1.p1 (https://www.ncbi.nlm.nih.gov/projects/gap/cgi-bin/study.cgi?study_id=phs000620.v1.p1). The data set includes the information from 2,183 families and 6,784 individuals. Supplementary Fig. 8 shows the QC process applied to this data set, resulting in a final total of 1,187 family trios used for the FT-SEM and lm_parent analyses. Moreover, lm uses the data of 2,183 independent individuals by randomly selecting one individual from each family. Additionally, the data set contains five phenotypes—NIC, CON, DEP, DRG and BD—all of which were used to carry out the GWAS.

The GWAS based on the MCTFR data set was conducted using three methods (FT-SEM, lm_parent and lm). When fitting FT-SEM, the offspring's gender, date of birth and age, and the father's and mother's dates of birth and ages were included as covariates. For lm_parent, we considered the covariates: the offspring’s gender, date of birth and age. lm incorporated the gender, the date of birth, the age and the generation of the individual as the covariates, and also took account of the interaction terms between generation and gender, generation and date of birth, and generation and age. Additionally, the first 10 principal components of all the autosomal SNPs were included in lm to adjust for population stratification.

To implement the two-sample MR analysis and investigate the causal effects of offspring’s BMI on offspring’s five phenotypes (NIC, CON, DEP DRG and BD), we first need to select IVs. Supplementary Fig. 9 details the filtering process of the IVs used in IVW FT-SEM and IVW lm_parent, while Supplementary Fig. 10 outlines the filtering process of the IVs for IVW lm. SNP-exposure results were derived either from the sibling-based GWAS^21^ (for IVW FT-SEM and IVW lm_parent) or from the GWAS summary data based on independent individuals^32^ (for IVW lm), while SNP-outcome results were obtained from our GWAS based on the MCTFR data set. Moreover, weak instruments with the F-statistic < 10 were excluded according to standard guidelines^37^. For IVW FT-SEM and IVW lm_parent, 27 independent SNPs ($r^{2}<0.001$ within 10,000 kb) significantly associated with BMI ($p<5\times 10^{-8}$) were selected. These SNPs were identified from the combined results of the sibling-based GWAS (“ieu-b-4816”) and the MCTFR-based GWAS using FT-SEM and lm_parent. For IVW lm, 448 independent SNPs meeting the same criteria were selected from the combined results of the independent GWAS (“ieu-b-40”) and the MCTFR-based lm. Additionally, the variants were clumped, and allele effect sizes were harmonized across the data sets using the TwoSampleMR package^38^. The weighted median, the weighted mode, and the MR-Egger are two-sample MR methods that are more robust to horizontal pleiotropy compared to IVW, but they have lower statistical power. Therefore, they can be used in sensitivity analyses to validate the results of our two-sample MR study. The summary data extraction, two-sample MR implementation, and sensitivity analyses (IVW, weighted median, weighted mode and MR-Egger) were performed using the TwoSampleMR package^38-40^.

## Data Availability

The Minnesota Center for Twin and Family Research data used for this study can be found on the database of Genotypes and Phenotypes with accession number phs000620.v1.p1 (https://www.ncbi.nlm.nih.gov/projects/gap/cgi-bin/study.cgi?study_id=phs000620.v1.p1.) The summary data from within-sibship GWAS are available at https://gwas.mrcieu.ac.uk/datasets/ieu-b-4816/. The summary data from independent GWAS are available at https://gwas.mrcieu.ac.uk/datasets/ieu-b-40/. The code using in this paper are available at https://github.com/ShunZhang0816/FT-SEM/.
